# Satellog: A database for the identification and prioritization of satellite repeats in disease association studies

**DOI:** 10.1186/1471-2105-6-145

**Published:** 2005-06-10

**Authors:** Perseus I Missirlis, Carri-Lyn R Mead, Stefanie L Butland, BF Francis Ouellette, Rebecca S Devon, Blair R Leavitt, Robert A Holt

**Affiliations:** 1 Genome Sciences Centre, BC Cancer Agency, Suite 100, 570 West 7th Ave, Vancouver, BC, V5Z 4S6, Canada; 2 UBC Bioinformatics Centre, University of British Columbia, 950 West 28th Ave, Vancouver, BC V5Z 4H4, Canada; 3 Centre for Molecular Medicine and Therapeutics, University of British Columbia, 950 West 28th Avenue, Vancouver, B.C., V5Z 4H4, Canada; 4 Department of Psychiatry, University of British Columbia, 2255 Wesbrook Mall, Vancouver, BC, V6T 2A1, Canada

## Abstract

**Background:**

To date, 35 human diseases, some of which also exhibit anticipation, have been associated with unstable repeats. Anticipation has been reported in a number of diseases in which repeat expansion may have a role in etiology. Despite the growing importance of unstable repeats in disease, currently no resource exists for the prioritization of repeats. Here we present Satellog, a database that catalogs all pure 1–16 repeat unit satellite repeats in the human genome along with supplementary data. Satellog analyzes each pure repeat in UniGene clusters for evidence of repeat polymorphism.

**Results:**

A total of 5,546 such repeats were identified, providing the first indication of many novel polymorphic sites in the genome. Overall, polymorphic repeats were over-represented within 3'-UTR sequence relative to 5'-UTR and coding sequence. Interestingly, we observed that repeat polymorphism within coding sequence is restricted to trinucleotide repeats whereas UTR sequence tolerated a wider range of repeat period polymorphisms. For each pure repeat we also calculate its repeat length percentile rank, its location either within or adjacent to EnsEMBL genes, and its expression profile in normal tissues according to the GeneNote database.

**Conclusion:**

Satellog provides the ability to dynamically prioritize repeats based on any of their characteristics (i.e. repeat unit, class, period, length, repeat length percentile rank, genomic co-ordinates), polymorphism profile within UniGene, proximity to or presence within gene regions (i.e. cds, UTR, 15 kb upstream etc.), metadata of the genes they are detected within and gene expression profiles within normal human tissues. Unstable repeats associated with 31 diseases were analyzed in Satellog to evaluate their common repeat properties. The utility of Satellog was highlighted by prioritizing repeats for Huntington's disease and schizophrenia. Satellog is available online at .

## Background

Anticipation is a medical observation that refers to the progressive worsening of a disease's symptoms and/or an earlier age of onset over successive generations of affected family members [1]. Although historically controversial, the concept gained widespread scientific acceptance with the identification in 1991 of unstable trinucleotide repeats associated with Fragile X syndrome [2,3] and spinal and bulbar muscular atrophy (SBMA) [4]. Today, 35 human diseases, some of which also exhibit anticipation, have been associated with unstable repeats [5]. Diseases for which unstable microsatellites are the causative disease mechanism can be divided into those caused by coding or non-coding repeat expansions.

The majority of disease-associated coding repeats identified to date are CAG-type repeats encoding an expanded poly-glutamine tract in affected individuals. CAG-type expansion disorders include spinal and bulbar muscular atrophy (SBMA) [4], dentatorubral-pallidoluysian atrophy (DRPLA) [6], Huntington disease (HD) [7] and a range of spinocerebellar ataxias (SCAs) including SCA1 [8], SCA2 [9], SCA3 [10], SCA6 [11], and SCA7 [12]. In these diseases, an expanded poly-glutamine tract results in a toxic gain of function causing either neuronal degeneration [13], or in mouse models of spinocerebellar ataxia (SCA), neuronal dysfunction due to Purkinje cell abnormalities [14]. The precise pathogenic disease mechanism is unknown but requires expression of the expanded polyglutamine tract. Neuronal inclusion bodies are observable on autopsy [14].

Untranslated repeats are diverse and include non-trinucleotide repeats. For example, progressive myoclonic epilepsy type 1 (EPM1) pathology results from an expansion of the dodecamer CCCCGCCCCGCG [15] and an ATTCT repeat expansion is the pathogenic agent in SCA10 [16]. In contrast to the coding repeat disorders, non-coding repeats can expand dramatically into the range of thousands of repeats [17]. Most non-coding repeat expansions are not associated with neuronal inclusion bodies on autopsy [14], with the exception of Fragile X-associated tremor ataxia syndrome [18], and nuclear foci observed in neurons of myotonic dystrophy patients [19].

Anticipation has been reported in a number of orphan diseases in which repeat expansion may have a role in etiology. These diseases include autosomal dominant limb-girdle muscular dystrophy [20], Crohn's disease [21], leukemia [22], nodal osteoarthritis [23], Parkinson's disease [24], rheumatoid arthritis [25], truncal heart defects [26], mood disorders [27], schizophrenia [28,29], and anxiety disorders [30,31]. Although no repeat expansions have been associated with any of these disorders, no comprehensive surveys have been undertaken.

Historically if one suspected a polymorphic microsatellite repeat were associated with a disease, few bioinformatics resources were available to identify relevant repeats in the human genome. One approach now available is to browse the Tandem Repeats Finder (TRF) [32] track on the UCSC genome browser [33] within a genomic region of interest. TRF at UCSC was executed with liberal insertion and deletion (indel) and substitution penalties that allow the detection of larger, frequently impure repeats. Since pure repeat tracts are more likely to expand than impure repeat tracts following transmission [34-36] a large fraction of repeats presented at UCSC are probably not relevant for disease association studies. Furthermore, certain known disease-associated repeats, such as the GAA repeat in Friedreich's Ataxia (chr9:67,109,320-67,109,339) [37], are not detected at all at UCSC because they are too short to be detected by their TRF parameters. Other groups have created databases of all 2–16 repeat unit satellite repeats within human gene regions [38,39] and of all 1–6 repeat unit microsatellites across prokaryotic and eukaryotic taxa [38]. Collins detected microsatellites with a novel algorithm and deposited this data in a relational database called GRID Short Tandem Repeats (STR) database [39]. This database included *in silico *polymorphism detection of coding trinucleotide repeats by using the BLAST algorithm to detect each repeat's length polymorphisms within GenBank, but only for a subset of coding repeats [39]. These resources enrich the microsatellite repeat bioinformatics landscape but do not integrate these data with other published resources in a way relevant for repeat prioritization in disease-association studies. Also, these resources do not provide flexible interfaces for combining data in user-defined ways to allow dynamic generation of candidate repeat lists. For example, both the Microsatellites Repeat Database (MRD) [38] and the STR databases [39] provide static co-ordinates of candidate repeats for disease-association studies defined by the author's criteria, but lack the functionality to easily re-prioritize repeats based on user preferences.

To address these deficiencies we created Satellog, a database that catalogs all pure 1–16 repeat unit satellite repeats in the human genome along with supplementary data we believe to be of use for the prioritization of satellite repeats in disease association studies. For each pure repeat Satellog can also calculate the percentile rank of its length relative to other repeats of the same class in the genome, its polymorphism within UniGene clusters [40], its location relative to known genes [41], and its expression profile in normal tissues according to the GeneNote database [42]. Repeats within Satellog can be prioritized based on any of their characteristics (i.e. repeat unit, class, period, length, length percentile rank, genomic co-ordinates), polymorphism profile within UniGene, proximity to or presence within gene regions (i.e. cds, UTR, 15 kb upstream etc), metadata of the genes they are detected within, and gene expression profiles within normal human tissues. Disease-associated repeats from 31 diseases were used as a test set to see what fraction could be detected independently within Satellog and what could be learned about polymorphic repeats in general. To showcase its utility, we used Satellog to prioritize repeats for disease-association studies in Huntington's disease and schizophrenia. Satellog is available as a web-queriable database along with all source code licensed under GNU General Public License at .

## Results

### Summary statistics

A total of 8,357,425 pure repeats were detected by TRF in the human genome and were stored in Satellog. Of these, 5,398,328 or 64.6% were detected within an EnsEMBL-defined gene or within 60 kb flanking either side of an EnsEMBL gene. These repeats mapped to 7,260,625 genetic locations in or near EnsEMBL genes, reflecting the fact that some repeats were located within more than one gene. Of the genes in EnsEMBL, 92% (21,654 / 23,531) had at least one pure repeat within 60 kb of their gene boundaries. All repeats in Satellog clustered into 70,318 unique repeat classes. Overall, repeat counts correlated with decreasing chromosomal size, however chromosome 19 had the highest density of repeats in accordance with previously published reports [43] (Figure S1, Table S1 – supplementary information available online at ). Data summarizing repeat counts and density by repeat unit size and chromosome (Table S2), by specific repeat unit (Table S3) and by gene region (Table S4) are also available online as supplementary information.

**Table 1:** Unstable coding repeats organized by descending standard deviation Sample output from Satellog.

**unit**	**length**	**gene location**	**pep**	**name**	**mean**	**sd**
**GCA**	**23**	**cds**	**LQQQQQQQQQQQQQQQQQQQQQQQ**	**AR**	**20.36**	**4.11**
**CAG**	**15**	**cds**	**QQQQQQQQQQQQQQQH**	**DRPLA**	**12.44**	**3.9**
GGC	17	cds	GGGGGGGGGGGGGGGGGE	AR	15.1	3.54
**CAG**	**19**	**cds**	**QQQQQQQQQQQQQQQQQQQQ**	**TBP**	**17.1**	**3.01**
ACC	13	cds	LPPPPPPPPPPPPP	NULL	11.5	2.12
GGC	8	cds	GGGGGGGGG	GDF7	9	1.73
CTG	6	cds	GSSSSSR	PCDH12	7.2	1.55
CCG	9	cds	PAAAAAAAAA	NULL	6	1.41
GGGGCC	4	cds	APAPAPAPAP	CDKN1C	3.33	1.15
GGC	6	cds	GGGGGG	NULL	6.67	1.03

The ten most unstable coding repeats organized by descending standard deviation. Repeats highlighted in bold are known disease-associated repeats. (Note: trailing non-consensus amino acids are not artefactual output. Repeat units continue to be detected at the DNA level even it they do not completely achieve the consensus. For example in the second row above, the corresponding DNA sequence (CAG)_15 _CA contains a trailing CA (of the subsequent CAT codon) that translates into histidine).

### Characteristics of disease-associated repeats

Disease-associated repeats and their common properties were recently reviewed [5]. We queried the database with these sequences to observe any characteristic features of these repeats relative to all other repeats. We asked how many of these repeats could be identified as potentially unstable using only the bioinformatics resources within Satellog. The co-ordinates for 31 of the 35 disease-associated repeats were manually collected from the review and identified in Satellog. Repeats that were not analyzed either had a repeat period greater than 16 (thus not detected by our TRF parameters) or were polymorphic but not associated with any disease. For these disease-associated repeats, there is no record of their precise genomic co-ordinates. To address this, we used Satellog to probe for the probable repeat that corresponded to each disease by selecting all repeats of the expected class within each disease gene. All repeats were detected, except for the repeat responsible for blepharophimosis [44]. In 12 cases, more than one candidate was detected as the disease-associated repeat for a disease. These cases usually involve flanking repeats of the same class that are detected as two distinct repeats because of an interrupting unit, an established characteristic of some disease-associated repeats such as those responsible for SCA1 [35] and Fragile X syndrome [36]. In these cases, we simply retained both repeats and associated them with the disease.

**Figure 1:**
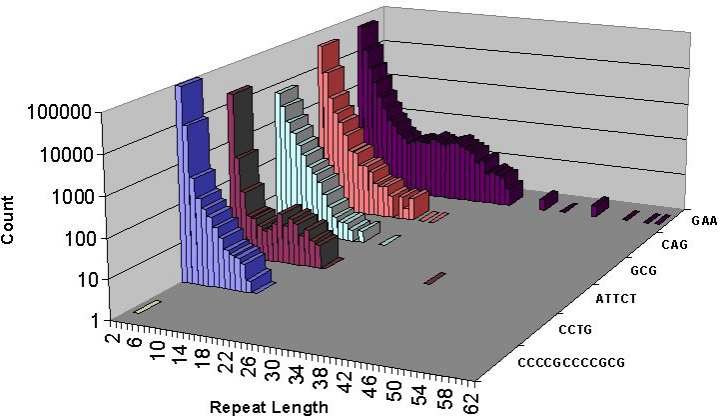
**Genome-wide repeat lengths of disease-associated repeat classes**. Genomic distribution of repeat lengths of all repeat classes associated with disease.

A total of 51 repeats were mapped for 31 diseases. Interestingly, these repeats were from only 6 repeat classes. Trinucleotide repeats are the most common repeat class implicated in disease [5], especially for disorders caused by coding repeat expansion. Of the disease-associated repeats we analyzed, 28 of the 31 were trinucleotide repeats with 16 being from the CAG repeat class, 11 from the GCG repeat class, and one each from the CCCCGCCCCGCG, CCTG, GAA, and ATTCT repeat classes respectively. These disease-associated repeat classes had dramatically different genomic distributions (Figure 1). For example, the CCCCGCCCCGCG dodecamer implicated in progressive myoclonic epilepsy type 1 (EPM1) [15] is the only pure repeat of its class detected in the human genome and therefore has a singleton as its distribution. The remaining repeat classes have broader distributions, particularly the GAA repeat class. GAA repeats have been reported to have a unique distribution relative to other trinucleotide repeats due to their evolutionary origin within *Alu *repeats [45]. Satellog recapitulated a distinct, expanded profile for GAA repeats relative to all other trinucleotide repeats (Figure 1).

We defined significant repeat length in the reference genome as any repeat with length within the top 5% of its class (corresponds to a percentile rank < 0.05 in Satellog). Using this cut-off, we determined whether the reference genome repeat length is significant for any of the disease-associated repeats within their respective disease classes. Interestingly, 80% (24/30) of the disease-associated repeats in Figure 1 were significantly long in the reference genome given their repeat class' length distribution (percentile rank < 0.05). In fact, 20 of 30 of all disease-associated repeats had a percentile rank of 0.01 or less indicating that these repeats were the extreme outliers within their class. Of the coding repeats, 12 of 17 had significant repeat lengths, including all the CAG-type repeats. Exceptions were the cleidocranial dysplasia (CCD), hand-foot-genital syndrome (HFGS), synpolydactyly, oculopharyngeal muscular dystrophy (OPMD), and holoprosencephaly coding GCG repeats. The CCCCGCCCCGCG dodecamer implicated in progressive myoclonic epilepsy type 1 (EPM1) is not included in this comparison because there were no other pure repeats of its class in the genome.

### Polymorphic repeats detected in UniGene clusters

**Figure 2:**
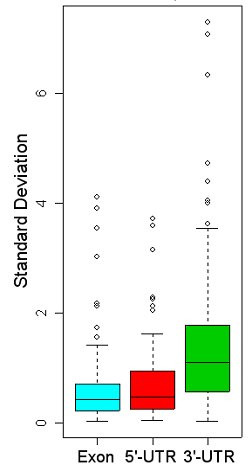
**Boxplot comparison of polymorphic repeats from coding, 5'-UTR and 3'-UTR sequence**. Median standard deviations (line through box) of all polymorphic repeats detected in coding, 5'-UTR, and 3'-UTR sequence. After controlling for sampling bias, coding and 5'-UTR standard deviations did not significantly differ from each other, but did significantly differ from 3'-UTR repeats implying that the 3'-UTR tolerates larger, more expanded repeats (*P *< 0.001).

**Figure 3:**
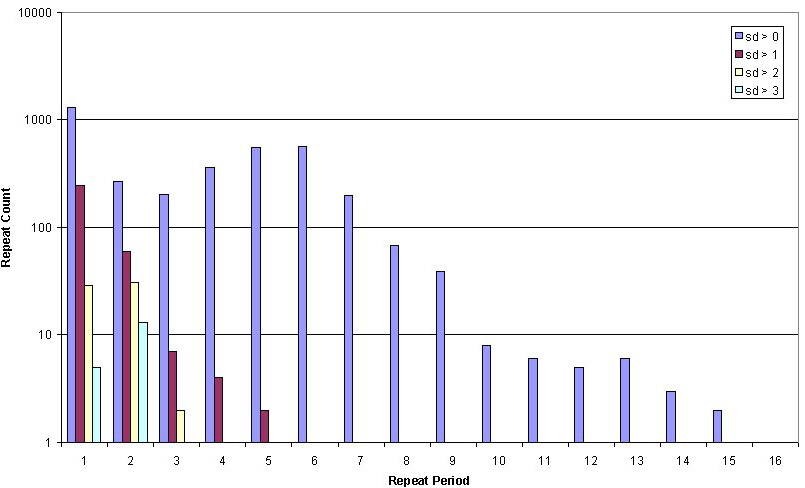
**Counts of unstable non-coding repeats at increasing instability cut-offs**. Repeat period distribution of polymorphic non-coding repeats at increasing standard deviation (sd) cut-offs.

**Figure 4:**
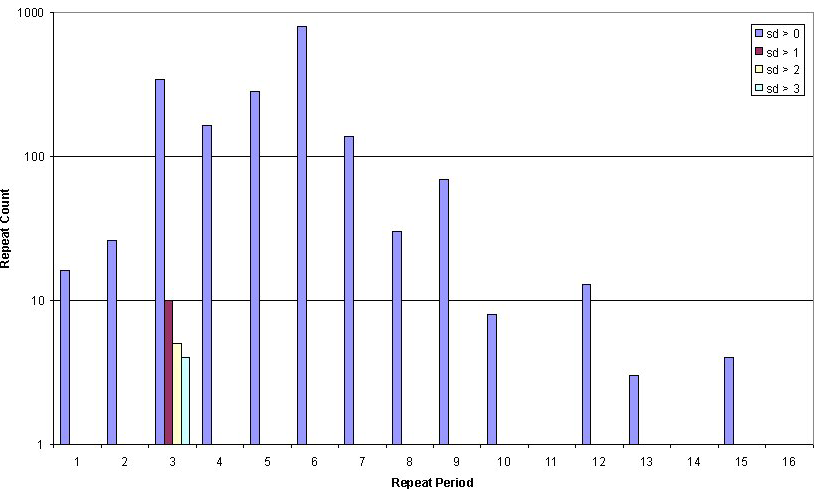
**Counts of unstable coding repeats at increasing instability cut-offs**. Repeat period distribution of polymorphic coding repeats at increasing standard deviation (sd) cut-offs.

We used a bioinformatics approach to see if we could detect repeat polymorphisms within UniGene sequences. Of the 8,357,425 pure repeats detected by Satellog, 1.3% or 111,950 repeats were detected as transcribed by the EnsEMBL API (either in the UTR or coding sequence (cds) of the gene). Of these repeats, approximately half (57.4% or 64,116 repeats) were detected within UniGene cluster sequences. Finally, of these repeats, only 5,546 repeats were detected as polymorphic (defined as any repeat that had at least one sequence within a cluster with a different repeat length). A measure of repeat polymorphism was provided by calculating the standard deviation (sd) of all repeat lengths detected within a UniGene cluster. A total of 2,763, 541, and 4,244 polymorphic repeats were detected in coding, 5'-UTR, and 3'-UTR sequence respectively (Note, repeats may exist in more than one gene which is why the location break-down of the repeats is greater than the total number of distinct polymorphic repeats of 5,546). Our ability to generalize repeat polymorphism trends within genetic regions was confounded by increased sampling of the 3' end of genes (Figure 2). To control for this, we compared the polymorphism profile of repeats in coding, 5'UTR, and 3'UTR regions that had equal sampling depth. By one-way ANOVA, we found a significant difference between coding (0.322 ± 0.134), 5'-UTR (0.416 ± 0.207), and 3'UTR (0.510 ± 0.184) repeats. There was significant repeat polymorphism in the 3'-UTR sequence relative to coding sequence but not to 5'-UTR sequence after controlling for sampling bias (Tukey-Kramer post-hoc multiple comparisons test, *P *< 0.001). Next we evaluated the tolerance of repeat polymorphisms by various repeat periods in coding and UTR sequence. To observe if highly polymorphic repeats were restricted to certain repeat periods (defined as repeat unit length), the repeat period distribution was observed at progressively increasing sd values (Figure 3 &4). Untranslated repeats were well distributed across all repeat periods except for 16 mers at an sd cut-off of 1 (which roughly corresponded to repeat polymorphisms of 1 repeat unit). At increasing sd cut-offs, untranslated polymorphic repeats were detected as penta-, tri-and mainly di-nucleotide repeats (Figure 3). In contrast, while coding repeat polymorphisms were widely distributed at an sd of 1, they were mainly restricted to trinucleotide repeats at higher sd cut-offs (Figure 4). Although the untranslated repeats had higher sd values, their most polymorphic sd values were restricted to mono-and di-nucleotide repeats.

### Disease-associated repeats detected in UniGene clusters

To address whether known disease-associated repeats were polymorphic within UniGene clusters, we extracted the top ten most polymorphic coding and non-coding repeats, based on their sd value, and determined if any of the disease-associated repeats were also the most polymorphic. The repeats associated with SBMA (*AR *is the gene mutated in individuals affected with SBMA), DRPLA, and SCA17 (*TBP *is the gene mutated in individuals affected with SCA17) were detected as the first-, third-and fourth-most polymorphic coding repeats (Table 1). The AIB-I repeat that confers increased risk of prostate cancer was also detected as polymorphic but not in the top ten. The repeat responsible for FRAXE was detected as polymorphic, but not as one of the top ten most polymorphic untranslated repeats (Table 2).

**Table 2:** Unstable untranslated repeats organized by descending standard deviation Sample output from Satellog.

**unit**	**length**	**gene location**	**name**	**mean**	**sd**
GT	9	3utr	NULL	12.17	7.29
AT	25	3utr	SPATA2	19	7.07
TA	10	3utr	NULL	11.11	6.33
T	11	3utr	LYZ	13.08	4.72
AC	23	3utr	NAV1	17.71	4.39
AC	28	5utr	NULL	25.4	3.71
GCA	16	5utr	GLS	9.6	3.58
GCC	14	5utr	DAZAP1	15	2.28
T	13	5utr	NULL	15	2.24
T	19	5utr	NULL	17.5	2.12

The ten most unstable untranslated repeats organized by descending standard deviation. No disease-associated repeats are present in this sample.

Of the 31 disease-associated repeats discussed previously, only 5 repeats were detected as polymorphic within UniGene clusters. We sought to understand why this occurred. Of the 31 disease-associated repeats, 4 failed to map within the genomic co-ordinates of any mapped UniGene cluster. The remaining 27 repeats mapped within a UniGene cluster's genomic co-ordinates. However, 16 of these failed to be detected within UniGene sequences even though they mapped within a UniGene cluster. This could be because of the 3' bias of the UniGene sequences, the incomplete nature of the clusters [40], sequence errors in the representative UniGene cluster sequence we searched against for hits (Hs.seq.uniq – see Methods for details), or the limitations of our mapping algorithm. Our approach enforces that the repeat must exist with at least 10 bp of flanking sequence, which leaves out repeats at the edge of UniGene clusters. The remaining 11 disease-associated repeats were detected within UniGene clusters, but only 5 of these repeats were polymorphic. On average, the repeats detected as polymorphic had more hits within UniGene clusters than those detected as stable (there were an average of 17.4 observations per repeat for the polymorphic repeats to 4.54 for stable repeats). This suggests that there is a greater chance of observing repeat polymorphism with deeper sampling. All of the polymorphic repeats were limited to one UniGene cluster and none of the lengths surpassed the disease pre-mutation threshold of 29, 25, 36, 42, and 39 pure repeats for the repeats responsible for increased prostate cancer risk (AIB-I), DRPLA, SBMA, SCA17, and FRAXE respectively [5].

## Discussion

Although one might expect greater polymorphism in UTR sequence relative to coding sequence due to reduced evolutionary constraints, both 5'-UTR and coding repeats had similar rates of polymorphism, whereas 3'-UTR repeats had significantly greater polymorphism compared to these two groups. This may be due to the documented 3'-UTR sequence over-representation in UniGene [40]. However, depending on whether the repeat is within coding or UTR sequence, there appears to be constraints regarding what repeat unit sizes can tolerate large polymorphisms. Of the more polymorphic UTR repeats (those with sd values greater than 3), there was a single trinucleotide repeat amongst mainly dinucleotide and mononucleotide repeats (Figure 2, Table 2). On the other hand, the majority of coding repeat polymorphisms, although less pronounced, are almost entirely in factors of three (Figure 1, Table 1). Our results support the observation that coding microsatellite polymorphisms are usually in-frame in order to avoid a deleterious phenotype resulting from frame-shift or to provide a rapid evolutionary response to a changing environment [46].

It is important to consider that larger repeat polymorphisms could cause a UniGene cluster to "split" into two distinct clusters. This could downplay a repeat's polymorphism because such repeats would not be evaluated as a single group, therefore decreasing the repeat's sd value. This issue was addressed by pre-mapping all UniGene clusters to the human genome. If the repeat co-ordinates were within 10 kb of the UniGene genomic co-ordinates, then the repeat length hits was retained and merged into a single sd value. In practical terms this was not an issue, since only one of our most polymorphic repeats (sd > 2) mapped to two clusters.

There are certain limitations in using the GeneNote database to establish expression of repeat-containing genes. Specifically, the GeneNote microarray experiments were conducted with whole tissues, not tissues from particular tissue sub-types [42]. For example, users limiting their search to repeats expressed in the brain must bear in mind the possibility that a transcript highly expressed in one anatomical region (i.e. hippocampus) may lack sufficient global expression to be detected in the whole brain tissue used by the GeneNote experiments. Users interested in expression in particular anatomical regions might benefit from integrating gene expression data from their anatomical region of interest with repeat data from Satellog.

**Figure 5:**
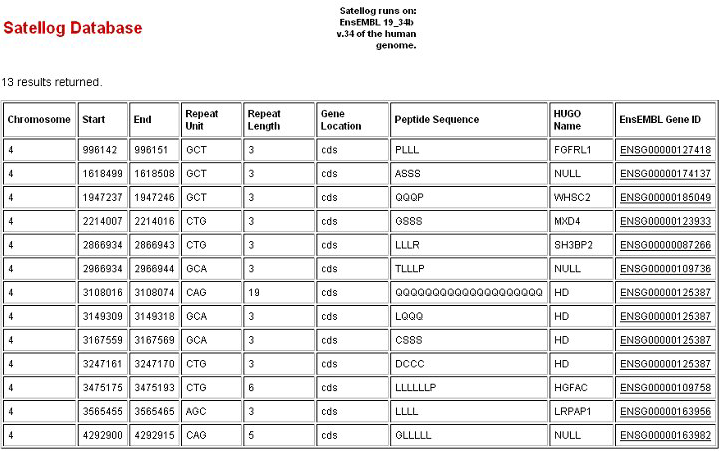
**Candidate repeats within Huntington's disease linkage region 4p16.3**. Sample output from Satellog summarizing candidate repeats within the 4p16.3 Huntington's disease linkage region. Coding CAG-type repeats from chr4:1-4,600,000 were selected along with their peptide sequence, HUGO names and ensembl gene IDs. The repeat encoding 19 glutamines has been associated with Huntington disease progression.

As an example of the utility of Satellog, we wished to see how it might have expedited research for groups in the past hunting for candidate unstable repeats. In 1992, haplotype analysis of linkage disequilibrium data in Huntington's disease patients had indicated a portion of 4p16.3 (chr4:1-4,600,000) as the likely location of the mutation [47]. We assumed that the investigators at the time were looking specifically for an unstable, brain-expressed, CAG repeat to explain the disease phenotype, similar to SBMA [4]. Using the Satellog database, we narrowed down our search for candidates repeats in this area from 13,804 to 13 (Figure 5). Three polyglutamine repeats are returned by the database, but the repeat implicated in Huntington's disease (chr4:3108016-3108074) stands out as a strong candidate due to its size. If we re-run this query and select only the top 5% of repeats relative to their class, chr4:3108016-3108074 is the only polyglutamine repeat. These repeat characteristics: CAG repeat type, brain expression and presence within the top 5% of its repeat class, plus the privilege of hindsight, easily allow us to distinguish this repeat as the lead candidate in this region.

Secondly, we sought to prioritize all repeats in disease in which unstable repeats might play a role but in which none have been successfully correlated with disease to date. Schizophrenia is one such disease with genetic linkage in region 22q [48-50] suggesting some role of chromosome 22 abberations in disease development. Microdeletions in this region in patients affected with Velocardial Facial Syndrome (VCFS) confers the most consistent genetic predisposition to developing schizophrenia [51]. First, we collected all repeats on chromosome 22 resulting in a total of 113,789 repeats. Next, since we only observed trinucleotide repeats and higher period repeats in our disease-associated set, we restricted our repeats to those with a period greater than 2 resulting in 91,918 repeats. Since the majority of the disease-associated repeats had a significantly longer reference genome length relative to other repeats of the same class, we selected the 2,934 repeats with a percentile rank less than 0.05. The cellular pathology associated with schizophrenia shows no evidence of nuclear inclusions mediated by polyglutamine expansions, therefore, the disease phenotype may be mediated by an expansion in the UTR region. We selected 27 repeats from our set that were located in either the 5'-or 3'-UTR. Assuming that genes relevant to schizophrenia are expressed in the brain, we limited our analysis to the 18 repeats that were within genes expressed in the brain. Of our final set of 18 repeats, 2 repeats in the 3'-UTRs of *CRKL *and *NIPSNAP1 *had evidence of repeat polymorphism in UniGene clusters (Table 3). In this prioritization paradigm, we did not look at any intronic repeats which may mediate the neurological phenotype by a mechanism similar to that of Friedreich's ataxia [37]. The point is that the prioritization paradigm can be defined by the user to dynamically generate a list of candidate repeats based on feature preference within Satellog or the fluctuating biological interpretation of repeat instability.

**Table 3:** Candidate repeats within the chromosome 22 schizophrenia linkage region.

**chr**	**start**	**end**	**unit**	**length**	**p-value**	**gene location**	**name**	**tissue**	**mean**	**sd**
22	19632267	19632294	AAC	9	0.019894	3utr	CRKL	Brain	8.04	0.51
22	28276064	28276078	GGCCT	3	0.017437	3utr	NIPSNAP1	Brain	2.97	0.17

Candidate repeats within the chromosome 22 linkage region implicated in schizophrenia along with the tissue expression call in the brain and UniGene cluster summary statistics indicating mean repeat length and polymorphism (standard deviation (sd) values > 0).

## Conclusion

Satellog enriches the current bioinformatics landscape in which repeats are viewed. For example, the GAA repeat in Friedreich's Ataxia [37] is not detected at all (chr9:67,109,320-67,109,339) in the UCSC genome browser [33] by the TRF [32] and Variable Number Tandem Repeats (VNTR) tracks. The VNTR feature in UCSC detects all perfect 2 to 10 repeat units with 10 or more copies. Repeats detected by this method may over-represent insignificant low period repeats and under-represent potentially interesting high period repeats. In Satellog, not only is the Friedreich's Ataxia GAA repeat detected, but its percentile rank also suggests that this size of GAA repeat is a relatively rare observation in the human genome (percentile rank = 0.045). Satellog integrates disparate data sources to give researchers an idea of how interesting certain repeats are based on their genetic location, tissue expression profile and polymorphism within UniGene. It should be noted that Satellog does not intend to be a *de novo *detection method for disease-associated repeats. Instead, it provides comprehensive, integrated bioinformatics platform to prioritize repeats in a convenient and efficient manner. Satellog also presents the first comprehensive identification and integration of disease-associated repeats with other genomic resources for use as bioinformatics reagents in other studies. Satellog should prove useful to investigators interested in prioritizing repeats for typing in diseases showing anticipation or in which repeat polymorphism is thought to play a role in etiology. In addition, given that all sequence information (i.e. the human genome sequence and UniGene sequences) is from presumed "normal" individuals lacking disease phenotypes; Satellog may also prove useful in extending our understanding of the normal role of repeats in genes and transcripts.

## Methods

### Software dependencies

A perl script "repeatalyzer.pl" functions as a wrapper for a number of different programs to achieve the endpoints of Satellog. repeatalyzer.pl is run with perl v5.6.1 and used BioPerl v1.2 [52], the EnsEMBL Perl API (May 24^th^, 1999 release), MySQL v10.8 Distribution 3.23.21-beta (for pc-linux-gnu), BLAT v. 28 [53] and v. 34 of the human genome sequence [54]. This script was run in parallel on a 192 node linux cluster at the BCCA Genome Sciences Centre. More detailed methods information is available at .

### Detecting microsatellite repeats with Tandem Repeats Finder (TRF)

We chose to detect sequences repeated at least twice and secondly, we were interested in exclusively pure repeat tracts which are more likely to expand following transmission [34-36]. Command-line TRF has seven parameters that can be manually assigned at run-time which include matching weight, mismatch and indel penalties, match probability, indel probability, minimum alignment score to report, and maximum period size to report [32]. We found that matching weight, mismatch and indel penalties, minimum alignment score and maximum period size directly affected the length and purity of hits detected by TRF whereas changing the match and indel probability features was not useful. The match and indel probability features refer respectively to the percent identity and fraction of indels tolerated in each serial tandem unit detected as a hit. These features allow users to specify alternative expected matching and indel statistical distributions.

Next we evaluated the ability of the matching weight and maximum period size parameters to detect short repeats. Period size refers to the length of the tandemly repeated DNA unit, for instance CAG repeats have a period of 3. Since TRF hits must be at least 10 bp, the smallest hit for each repeat class reported in Satellog is 10 divided by the repeat unit length. For example, for CAG repeats, the smallest hit detectable that satisfies the minimum hit length is a 3 1/3 repeat unit hit (i.e. CAG CAG CAG C). In short, only pentanucleotide and larger repeats have a minimum of two repeat units in Satellog.

Lastly we investigated the utility of adjusting the mismatch and indel penalties. We found that setting the penalty for these parameters to 4090 produced no impure repeats as hits. TRF was run on whole chromosome FASTA files from v. 34 of the human genome downloaded from the UCSC genome browser. Hit purity was confirmed by visually inspecting the top high period hits (these hits have the highest probability of introducing indels due to the scoring scheme used by TRF [32].

### Identifying unique repeat classes

A repeat can be represented in a number of ways in double-stranded DNA. TRF detects repeats by the first tandemly repeated unit, therefore, CAGCAGCAG, AGCAGCAGC, and GCAGCAGCA are detected as repeats of CAG, AGC, and GCA respectively. Furthermore, the reference human genome sequence is only presented as the positive strand. Repeats of GTC, TCG, and CGT on the positive strand represent 5'->3' CAG, AGC and GCA repeats respectively on the negative strand. Therefore, to identify all CAG repeats in the human genome it's necessary to detect all CAG, AGC, GCA, GTC, TCG, and CGT repeats on the positive strand. We developed an algorithm to generate all possible sequence varieties of a repeat unit on the positive and negative strands. Our repeat classification algorithm operates by taking an input repeat unit, i.e. CAG, removing the first letter (C in this case) and appending it to the end of the remainder (AG) to create the second repeat unit (AGC). This is then reverse complemented to generate the equivalent sequence on the negative strand (TCG). This procedure is repeated repeat unit length – 1 times to generate a unique identifier henceforth referred to as the repeat class. Each repeat in Satellog is associated with a single unique repeat class.

### Preparing AffyMetrix expression data from the GeneNote database

The GeneNote (Gene Normal Tissue Expression) database provides baseline normal expression data of human genes for use in disease studies [42]. GeneNote data is downloaded from the Gene Expression Omnibus (GEO). A total of twelve human tissue profiles are presented in GeneNote including bone marrow, brain, heart, kidney, liver, lung, pancreas, prostate, skeletal muscle, spinal cord, spleen, and thymus. These products were generated with the AffyMetrix HG-U95 A-E probe-set, covering 62,839 probe-sets. EnsEMBL genes have been mapped to AffyMetrix HG-U95 probes by the EnsEMBL project [41]. Once a repeat is detected either inside or within 60 kb of an EnsEMBL gene, that gene's normal expression profile is evaluated by cross-referencing its AffyMetrix tags to the GeneNote database within Satellog.

### Detecting repeat polymorphisms within UniGene clusters

UniGene contains the largest public repository of transcribed human sequence and represents an attempt to organize this wealth of expression data into discrete transcriptional loci [40]. All human UniGene sequences were processed for use with repeatalyzer.pl. For each repeat detected in UTR or coding sequence, the repeat plus 10 bp of flanking sequence was extracted from EnsEMBL and queried using the BLAT algorithm [53] against a BLAT-formatted database created from sequences representing the longest, highest quality stretch of DNA from each individual UniGene cluster (pre-selected by UniGene as the file Hs.seq.uniq). Polymorphism is evaluated only if BLAT analysis against all UniGene clusters resulted in 1) hits that achieved BLAT scores at least 85% of the theoretical maximum for a perfect hit 2) 90% of the query sequence matched identically within the cluster 3) the repeat mapped within 10 kb of the genomic co-ordinates of the UniGene cluster. If a hit to a UniGene cluster satisfied these criteria, the length of the repeat in the cluster is stored in Satellog. This feature allows investigators to query all repeats with polymorphisms in UniGene clusters from genomic regions of interest.

### repeatalyzer.pl overview

**Figure 6:**
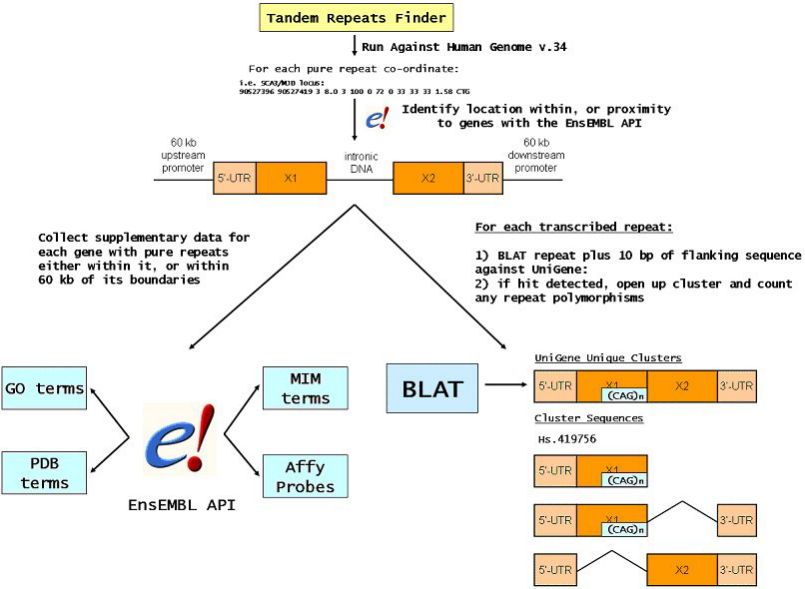
**repeatalyzer.pl flowchart**. Flowchart outlining how repeatalyzer.pl populates the Satellog database.

Once the above software and data dependencies are configured, repeatalyzer.pl automatically populates Satellog (Figure 6). The script processes the flat files output by TRF. These files contain the repeat co-ordinates plus the repeat period (the size of the repeated unit), the sequence of the individual repeat unit, the entire repetitive sequence and the repeat length. Repeat co-ordinates are passed to the EnsEMBL API to confirm the authenticity of the co-ordinates generated by TRF. If the repeat is not detected within a gene with the EnsEMBL API, then progressively larger slices incrementing by 15 kb are taken in search of flanking genes. As soon as a gene is located in flanking sequence then no further flanking sequence is collected. However, if no genes are detected within 60 kb of the repeat co-ordinates then repeatalyzer.pl stops searching for genes. If a repeat is detected inside or within 60 kb adjacent to an EnsEMBL-defined gene then that gene's primary information (co-ordinates, HUGO name, EnsEMBL ID and description) are collected along with metadata stored in EnsEMBL such as Protein Data Bank (PDB) [55], Online Mendelian Inheritance in Man [40], Gene Ontology (GO) [56], and mappings to AffyMetrix probe sets. If the repeat is located in the 5'-UTR, 3'-UTR, or coding sequence of a gene then its polymorphism profile within UniGene clusters is evaluated.

### Generating a measure of repeat length significance

After running the script to populate Satellog, each repeat's length is compared to the lengths of all repeats of the same repeat class. The majority of repeats associated with disease undergo expansions from already large reference genome lengths relative to other repeats of the same class [5]. Each repeat's percentile rank is calculated from the distribution of repeat lengths within each repeat's class. It reflects the proportion of repeats with the same or greater length from the repeat class' genomic distribution.

## Authors' Contributions

PIM conceived of the study, wrote all analysis scripts, collected and input data into the database, analyzed the data, directed the Satellog website design, wrote all documentation and the tutorial accompanying the database and drafted the manuscript. CRM developed the online graphical user interface for the database, troubleshooted and re-indexed queries for the database and provided technical expertise for realizing the web version of Satellog. SLB participated in the design of the study and gave crucial intellectual direction to the final manuscript. BFFO participated in the design of the study and provided assistance with bioinformatics analysis. RSD provided key biological background to guide the design of the study. BRL participated in the design and strengthened the clinical perspective of the final manuscript. RAH participated in the study design, coordination, performed data analysis and gave critical direction to the final manuscript. All authors read and approved the final manuscript.

## Appendix

Figure S1 – Repeat density (bp of repeat sequence / Mb) per human chromosome.

Available online at: .

Table S1 – Total repeat count and density by chromosome

Available online at: .

Table S2 – Repeat period count and density by chromosome

Available online at: .

Table S3 – Repeat unit count and density by chromosome

Available online at: .

Table S4 – Repeat unit count and density by gene region

Available online at: .
